# Infoveillance of COVID-19 Infections in Dentistry Using Platform X: Descriptive Study

**DOI:** 10.2196/54650

**Published:** 2025-04-03

**Authors:** Alghalia Al-Mansoori, Ola Al Hayk, Sharifa Qassmi, Sarah M Aziz, Fatima Haouari, Tawanda Chivese, Faleh Tamimi, Alaa Daud

**Affiliations:** 1 College of Dental Medicine QU Health Qatar University Doha Qatar

**Keywords:** COVID-19, dentistry, infection, patient, infoveillance, platform X, Twitter

## Abstract

**Background:**

The effect of the COVID-19 pandemic on the well-being of dental professionals and patients has been difficult to track and quantify. X (formerly known as Twitter) proved to be a useful infoveillance tool for tracing the impact of the COVID-19 pandemic worldwide.

**Objective:**

This study aims to investigate the use of X to track COVID-19 infections and deaths associated with dental practices.

**Methods:**

English Tweets reporting infections or deaths associated with the dental practice were collected from January 1, 2020, to March 31, 2021. Tweets were searched manually using the X Pro search engine (previously known as TweetDeck [X Corp], Twitter Inc, and TweetDeck Ltd) and automatically using a tweet crawler on the X Academic Research application programming interface. Queries included keywords on infection or death of dental staff and patients caused by COVID-19. Tweets registering events on infection or death of dentists, dental staff, and patients as part of their conversation were included.

**Results:**

A total of 5641 eligible tweets were retrieved. Of which 1583 (28.1%) were deemed relevant after applying the inclusion and exclusion criteria. Of the relevant tweets, 311 (19.6%) described infections at dental practices, where 1168 (86.9%) infection cases were reported among dentists, 134 (9.9%) dental staff, and 41 (3.1%) patients. The majority of reported infections occurred in the United States, India, and Canada, affecting individuals aged 20-51 years. Among the 600 documented deaths, 253 (42.2%) were dentists, 22 (3.7%) were dental staff, and 7 (1.2%) were patients. The countries with the highest number of deaths were the United States, Pakistan, and India, with an affected age range of 23-83 years.

**Conclusions:**

The data suggest that analyses of X information in populations of affected areas may provide useful information regarding the impact of a pandemic on the dental profession and demonstrate a correlation with suspected and confirmed infection or death cases. Platform X shows potential as an early predictor for disease spread. However, further research is required to confirm its validity.

## Introduction

As a global pandemic, COVID-19 has affected people from more than 200 countries and regions, leading to more than 500 million confirmed cases and more than 6 million deaths as of June 2022 [1,2]. COVID-19, caused by SARS-CoV-2, is thought to spread via close contact through respiratory droplets and aerosols. Due to aerosol generation at the dental chair by air-turbine handpieces, as well as the proximity to patients during dental procedures, dentistry was thought to be associated with the spread of infection. On the other hand, the identification of SARS-CoV-2 in the saliva of infected patients, and the possible transmission of COVID-19 through asymptomatic carriers, posed a threat to the dental profession [1-3].

Dental professionals were at an increased risk of infection, prompting extensive revisions in practice protocols to ensure safety. Practices adopted rigorous infection control measures, including the use of personal protective equipment, enhanced disinfection procedures, and the introduction of tele-dentistry services to reduce physical visits [3]. Statistically, health care workers, including those in dental settings, experienced considerable infection rates and mortality. A systematic review highlighted that, globally, more than 150,000 infections and around 1400 deaths occurred among health care workers by early May 2020. The review pointed out that while health care workers composed a substantial proportion of COVID-19 cases, the specific impact on dental professionals required further detailed study to fully understand the nuances and risks involved [4].

The pandemic led to significant changes in dental practice operations. Nonemergency procedures were postponed and dental clinics were urged to treat only urgent conditions to minimize the risk of virus spread. These changes were based on guidance from leading dental and health organizations, which continuously adapted their recommendations in response to the evolving pandemic situation [5]. Therefore, it was crucial to identify the risk of bidirectional spread of infection between patients and dental professionals to take additional precautionary measures to halt the spread of COVID-19. During the period of the pandemic, countries experiencing the spread of infection had to suspend routine dental care and deliver only urgent care with heightened cross-infection controlled measures [6,7]. With the explosion of information on social media, it proved challenging to find guidance and reliable research evidence on the nature and impact of COVID-19 in relation to dentistry.

Social media has increasingly become an important source of information. Throughout the pandemic, social media played an active role in the exchange of oral health and dental-related information. In addition to shaping the way health-related information spreads, and enhancing communication modes between health care providers and patients, social media has also attracted the attention of researchers to study the distribution of diseases, public reactions to health events, and dissemination of information and misinformation [7-10]. Examples of social media platforms include YouTube, Weibo, LinkedIn, and Facebook [7,9,11]. Among the different social media platforms, X (previously known as Twitter) is one of the most popular forms used for health care communication [11].

In the field of medicine, platform X (formerly known as Twitter) has been used as a source of social media data within the context of health care–related information [11]. X allows millions of users to send and receive “tweets” or short messages for free. The networking and microblogging service has nearly 400 million registered users worldwide and processes about 2 billion tweets per day [12]. Much useful information and recent news events have been documented via X directly from users at the site in real time [13]. The year 2020 was considered a “transformative” year, where X data showed that hashtags about coronavirus were the most popular, followed by social injustice themes and stay-at-home hashtags [14].

Predicting the number of suspected or confirmed cases of COVID-19, the spread of infection, and its implications are crucial in the prevention and control of virus outbreaks. An “infoveillance” approach using X has been proposed in the literature to measure public perceptions and track levels of disease activity during the pandemic [13,15-17]. Even though Platform X proved to be a useful tool for tracking the impact of the COVID-19 pandemic worldwide, data on the effect of COVID-19 on the dental profession remains scarce [18]. Accordingly, to explore the number of suspected or confirmed cases of COVID-19 within the dental profession worldwide, the current descriptive study aims to investigate reported COVID-19 infections and deaths in dental practices, including patients, dentists, and dental personnel.

## Methods

### Study Design and Search Strategy

**Figure 1:**
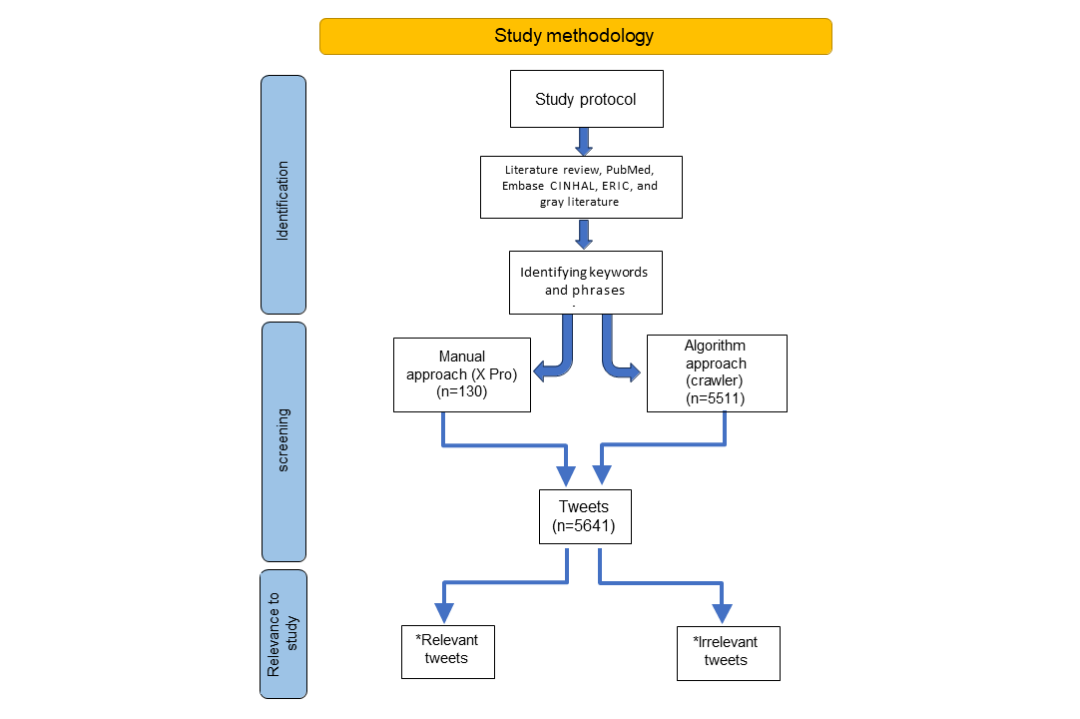
Study methodology. Relevant tweets are related to dental professionals and dental patients, reporting an infection/death case among dental professions and patients, first-hand witness reports, and verified X account. Irrelevant tweets include, for example, advert tweets, repeated tweets (retweeted), or reporting the same case.

A protocol was established to outline the study’s objectives and methodologies, ensuring process transparency. This protocol specified the inclusion and exclusion criteria for evidence review, identified relevant data, and described the methods for data extraction and presentation (Figure 1).

The research protocol involved a literature review to identify studies related to the topic followed by constructing the appropriate methodology to extract relevant tweets. A literature review was conducted to explore research investigating the use of X to predict the number of new COVID-19 cases and track the impact on dentistry. The inclusion criteria listed in Textbox 1 were applied:

**Inclusion criteria.**

**Inclusion criteria**

- Tweets in English language.
- Clear infection or death location.
- Firsthand witness, personal experience, news outlet or government, announcement, and professional organization.
- Clear COVID-19 infection or death of dental patients, dentists, dental staff, and dental students.
- Date of infection or death from the start of the pandemic to the end of March 2021.
- The location of the tweet is clear.

The following were excluded: repeated tweets or retweets, no clear number of infections or deaths, the tweet disappeared, or not a trusted source, and retired dental professionals.

January 1, 2020, was chosen as the start date for the search, as this was when COVID-19 was officially reported to the World Health Organization (WHO) from Wuhan. The term “coronavirus” was only formally announced in February. However, this was taken into consideration when screening tweets describing COVID-19–like symptoms.

Keywords were selected through titles, abstracts, full texts, and their references. Full-text papers were accessed via institutional subscriptions, Google Scholar, ResearchGate, or by directly contacting the authors. Exclusions were applied to discussion papers and letters to the editor. Searches across databases used Boolean operators “AND” and “OR,” along with truncation and phrase search techniques. The review aimed to catalog both published and gray literature pertaining to the topic from January 2020 to January 2022, encompassing the COVID-19 pandemic period. Searches were conducted in scientific databases including PubMed, Embase, and CINAHL, as well as educational databases such as the Education Resources Information Center and ProQuest. A supplementary search was performed using Google Scholar. Keywords included, but were not limited to, COVID-19 [tw] OR “COVID 19” [tw] OR SARS2 [tw] OR “SARS 2” [tw] OR SARS-COV-2 [tw] OR Coronavirus [tw] OR “severe acute respiratory syndrome coronavirus 2,” dental [tw] OR dentistry [tw] OR dentist* [tw].

### Data Collection

**Figure 2:**
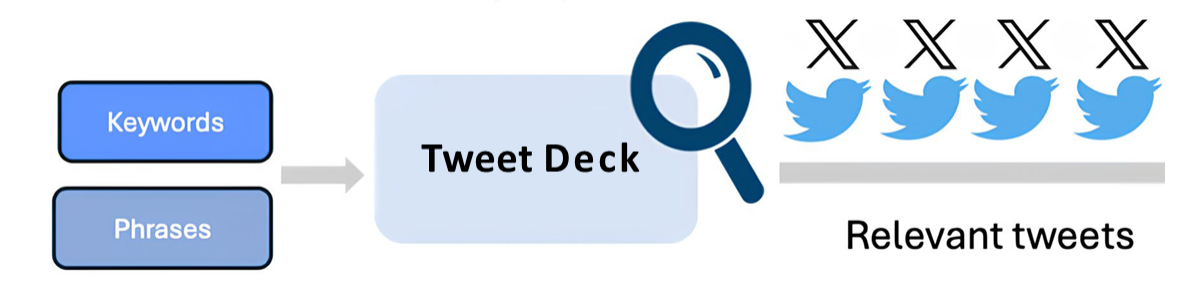
Manual approach using X Pro (previously known as TweetDeck) to extract tweets.

To collect the tweets, authors AD, OA, AA, and SQ created new X accounts at the start of the study to avoid having any search histories or previous likes that may promote preferential links. Two approaches were adopted for the tweet search. The first was a manual approach using X Pro (previously known as TweetDeck), a user interface provided by X that allows searching tweets given some keywords or phrases as shown in Figure 2. This approach was used based on evidence from the literature [7]. AD, OA, AA, and SQ independently conducted the search, collected the tweets, and stored them. All relevant information was stored on a Microsoft Excel sheet on a password-protected computer. Tweet texts were copied onto the Microsoft Excel sheet and saved. The manual approach was conducted first as the initial results were then used as a pilot to develop the search and pave the path for the second approach [7,19,20]. It served as a way to gain insights into the available literature and refine the search strategy for subsequent searches. By examining the initial results, researchers could identify relevant keywords, sources, and inclusion criteria, which could then be used to develop a more systematic search approach. This iterative process ensured that the final search strategy was comprehensive, targeted, and aligned with the research objectives [21].

**Figure 3:**
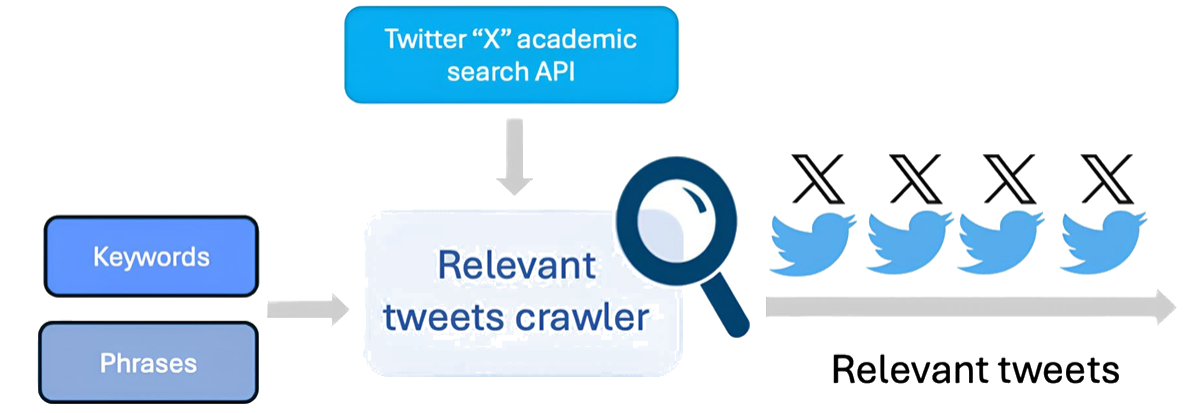
Automatic approach using a tweets crawler (X search algorithm) to extract tweets. API: application programming interface.

The second is an automatic approach using a tweets crawler (X search algorithm) as shown in Figure 3, which is implemented using Python programming language (Python Software Foundation) and exploits the X Academic Research Application Programming Interface (API) [19]. The API is relatively new and provides free access to the full history of public tweets via the full-archive search endpoint. Several studies exploited the API recently to collect historical COVID-19–related tweets, where they targeted vaccine-related tweets [19-21]. Differently, our focus is dental-related tweets. To collect the tweets, 2 queries were provided as input to our crawler as shown in Table 1. The queries include English keywords either about the infection or about the death of dental personnel and patients caused by COVID-19. We adopted the inclusion-exclusion principle offered by the API, for example, (dental OR dentist OR dentists) AND (death OR deaths) AND (infection OR infections) AND (COVID OR COVID-19 OR Corona). Since the API makes it possible to construct queries up to 1024 characters, we were able to search using long queries where we tried to include all possible keywords used in dental care, for example, dental hygienist, dental therapist, dental nurse, or dentist patient, to name a few. The mentioned queries were decided based on discussions with dentistry experts and the authors of the paper, along with insights from a pilot study that reviewed a sample of retrieved tweets to finalize the queries, as well as results from the initial manual search.

**Table 1:** Two queries were used as inputs for the crawler search.

Query	Inputs
Infected dentist’s, patients, and staff query	(dental OR dentist OR dentists OR dental surgeon OR dental clinic OR dental staff OR dental therapist OR dental nurse OR endodontist OR exodontist OR orthodontist OR pedodontist OR periodontist OR prosthodontist OR dental hygienist OR dentist hygienist OR dental assistant OR dentist assistant OR dental receptionist OR dentist receptionist OR dentist patient OR dental patient OR dentist patients OR dental patients) AND (positive OR infected OR infection) AND (covid OR covid-19 OR covid 19 OR covid19 OR corona OR coronavirus OR SARS2 OR SARS-2 OR variant covid virus OR variant coronavirus OR new strain covid OR covid vaccination OR corona vaccination)
Dead dentists, patients, and staff query	(dental OR dentist OR dentists OR dental surgeon OR dental clinic OR dental staff OR dental therapist OR dental nurse OR endodontist OR exodontist OR orthodontist OR pedodontist OR periodontist OR prosthodontist OR dental hygienist OR dentist hygienist OR dental assistant OR dentist assistant OR dental receptionist OR dentist receptionist OR dentist patient OR dental patient OR dentist patients OR dental patients) AND (death OR deaths OR fatality OR fatalities OR passed away OR pass away OR mortality OR mortalities OR died OR succumb OR decease OR deceased OR perish OR perished OR rest in peace) AND (covid OR covid-19 OR covid 19 OR covid19 OR corona OR coronavirus OR SARS2 OR SARS-2 OR variant covid virus OR variant coronavirus OR new strain covid OR covid vaccination OR corona vaccination)

Using the automatic approach, we retrieved the tweets posted from January 1, 2020, to March 31, 2021. To avoid retrieving duplicate tweets we configured our crawler to exclude retweets, however, we kept replies as users can mention events about the infection or death of dental staff, and patients as part of their conversation about similar events, or to confirm the event mentioned in the original source tweet. While automatically retrieving the tweets we excluded those that we already retrieved by the manual approach.

### Data Screening

Data retrieved related to tweets were screened. First, tweets were divided into “relevant” and “irrelevant” tweets. Irrelevant tweets did not mention a case of death or infection of the dentist, dental personnel, or dental patient. While relevant tweets mentioned a case of death or infection of the dentist, dental personnel, or dental patient. Second, relevant tweets were divided into included and excluded tweets.

Information in tweets was retrieved including the location of the tweet, the location of reported infection or death, the age, gender and the number of patients, dentists, or dental personnel referred to, the reported reason for infection and the outcome, the source, ID and link of the tweet, and the full text of the tweet. Tweets were scrutinized to whether they indicated a COVID-19 infection or death or were merely an acknowledgment of the author’s awareness of COVID-19, whether the infection was in reference to the author or another person, and whether it seemed a genuine report or speculations. Repeated tweets were initially included and then excluded when data were filtered. Tweets mentioning the same dentist, nurse, dental personnel, or patient name were also considered included during the first stage, then excluded when calculating the final numbers. In addition, tweets that mentioned the same hospital, clinic, or location at the same date and time or using the same link as a reference for the infection or death were also excluded.

### Data Analysis

Reports from tweets were analyzed descriptively. A total of 3 team members conducted the descriptive analyses, which included creating tables and graphs. Narrative analyses were also performed to describe events by location, type of dental personnel, and other relevant characteristics.

The number of infections and the number of deaths were described by country. The number of infections and deaths in each country was also compared with the number of WHO-registered dentists in that country. Data from the retweets were not included in the counts of infections and deaths.

### Ethical Considerations

No Institutional Review Board Ethics approval was required per Qatar University’s Institutional Review Board (QU-IRB) guidelines as all data were publicly available and deidentified, and no personal or confidential information was used (22).

## Results

During the 15 months of data collection, a total of 5641 eligible tweets were retrieved. Of which 1583 were deemed relevant after applying the inclusion and exclusion criteria. Tweets retrieved from both X Pro and Crawler were analyzed (Table 2). Of the relevant tweets, 311 described infections at dental practices, where 1168 infection cases were reported among dentists, 134 dental staff, and 41 patients. Of these, 30 were males, 43 were females, and the gender of the remainder was not reported. The most common countries reporting COVID-19 infections on X were the United States, India, and Canada, with an age range of 20-51 years.

**Figure 4:**
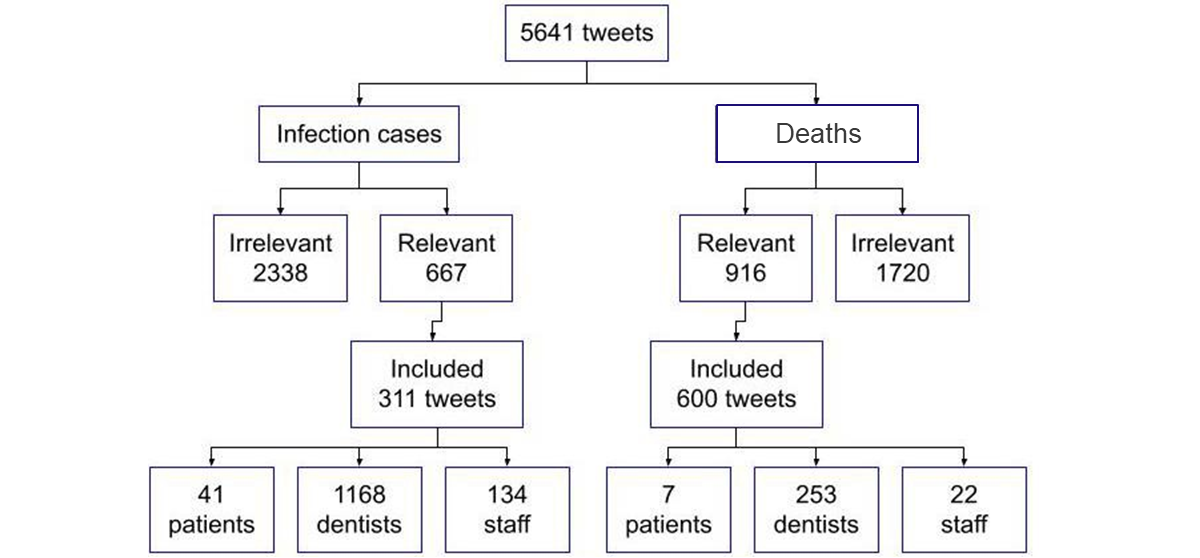
Relevant and irrelevant number of tweets reporting COVID-19 infections and deaths by users on X.

Out of the 916 relevant tweets, 600 deaths from COVID-19 were described via X users, of which 253 were among dentists, 22 among dental staff, and 7 among patients (Figure 4). Of these, 98 were reported as males and 32 as females, and the gender of the remainder was not specified. The most common countries reporting deaths due to COVID-19 infections were the United States, Pakistan, and India, with an age range of 23-83 years.

**Table 2:** Number of tweets retrieved through X Pro and Crawler approaches.

X platform/extracted information	X Pro	X search algorithm (Crawler)
Search method	Manual approach	Automatic approach
Number of tweets	130	5511
Number of irrelevant tweets	0	4058
Number of relevant tweets	130	1453
Number of included tweets	76	835
Number of excluded tweets	54	618
Number of final included tweets	63	288

**Figure 5:**
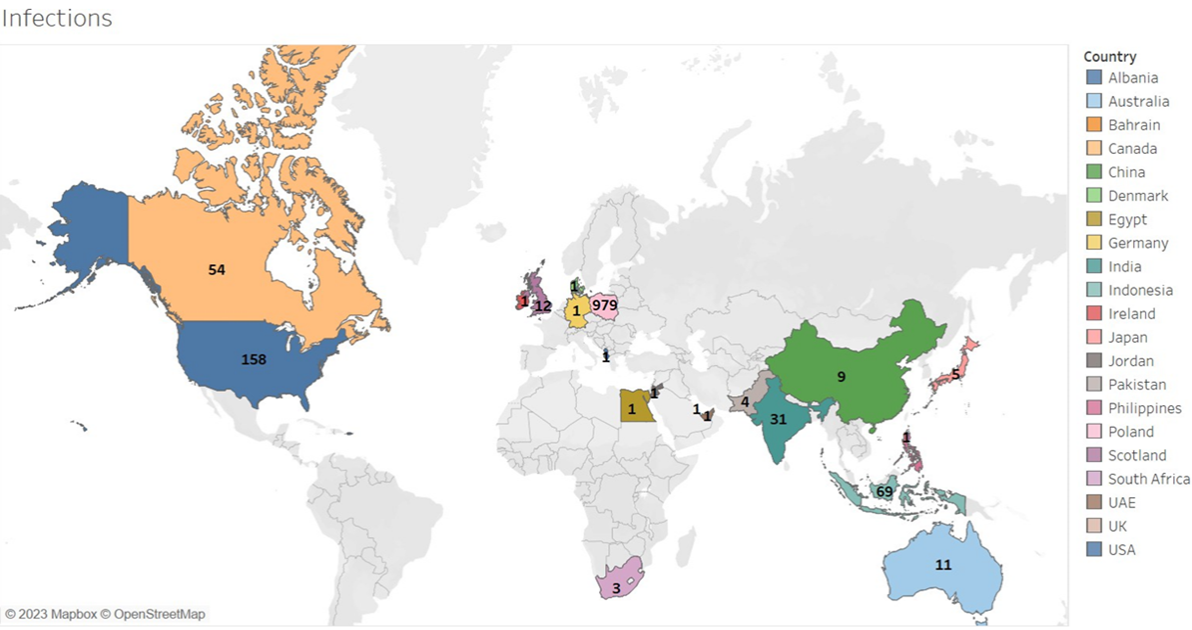
Map showing tweets by users reporting the infection of dentists, dental staff, or dental patients worldwide. Map data from OpenStreetMap [24].

**Figure 6:**
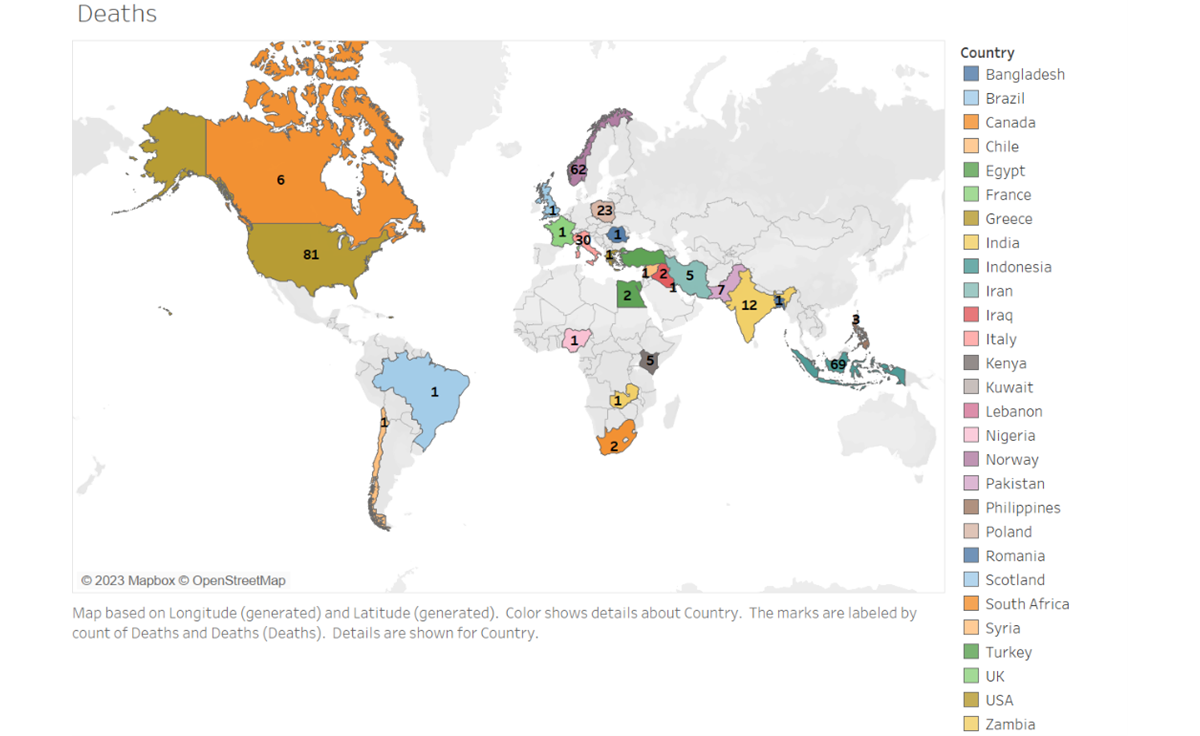
Map showing tweets by users reporting the death of a dentist, dental staff, or dental patients worldwide. Map data from OpenStreetMap [24].

Analyses of the tweets were performed, and geographical landmarks were outlined on a map. An example of a map showing the number of tweets by users reporting the infection and death of a dentist, dental staff, or dental patients worldwide is given in Figures 5 and 6. An important aspect of accurate social media surveillance is identifying the geographic location of each tweet [23]. To estimate COVID-19 prevalence in a geographic region, only tweets from that region were included, while tweets with an unclear country, state, county, or city were excluded. The map presenting the geographical data was created using Tableau (2022 Tableau), an internet-based software for building several different types of maps for geographic analysis. For example, the number of death cases was entered along with the corresponding location [24]. It was not possible to retrieve X data from certain countries that do not use this particular form of social media and may be using alternative platforms, such as China, Iran, and Russia.

The current descriptive study noted that a large number of COVID-19–related oral health information was tweeted during the pandemic, and people were reacting quickly to the emerging infection. Public reactions in tweets ranged from warning of infected dental personnel in a specific clinic, describing the possible risk of contracting the infection during a dental procedure, the rapid spread of COVID-19 in clinics where there was no lockdown applied, and much more.

**Figure 7:**
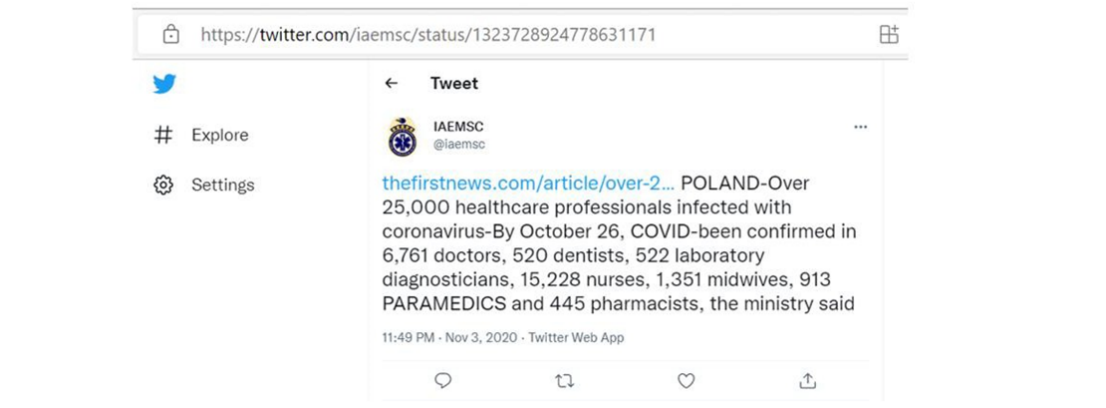
A tweet confirming COVID-19 infection in 520 dentists in Poland.

Some tweets from trusted sources reported a large number of confirmed cases in particular regions. Therefore, the number of COVID-19 cases in this study outlines the number of tweets. An example of such tweets can be found in Figure 7.

The tweets from each country were also compared with data from the WHO and relevant government sources to identify any correlation with confirmed infection cases or deaths. However, no direct correlation was identified as tweets do not always describe or include patient demographics or characteristics. To explore the rate of infections and deaths among dentists as reported on platform X, the number of registered dentists in each country was also investigated and compared with the reported cases on X (Table 3). Analysis of retweets showed no significant difference from the original tweets, showing little benefit in including second-hand personal information or personal experiences [17]. Therefore, retweets were excluded from this study.

**Table 3:** Data from the World Health Organization (WHO) and relevant government sources on the number of infections, deaths, and the total number of registered dentists.

Location of infection	Number of infections in the location	Number of deaths in the location	Dentists number (WHO) Last Update (between 2016-2021)
Germany	1	0	71,108 in 2020
Bahrain	1	0	134 in 2016
Canada	44	6	24,909 in 2020
United States	33	63	201,900 in 2021
United Kingdom	5	4	34,673 in 2021
Philippines	1	2	28,378 in 2021
Pakistan	3	4	26,686 in 2019
Japan	0	0	104,152 in 2020
Kenya	0	5	1300 in 2019
India	17	9	222,816 in 2020
South Africa	2	2	6586 in 2021
Egypt	1	2	19,746 in 2018
Jordan	1	0	7917 in 2019

Table 4 also shows a comparison between data reported in tweets and data from the Centers for Disease Control and Prevention (CDC) during 2020 [26,27]. A total of 192 tweets were identified from the United States reporting 117 Infections among dental professionals, slightly higher than the data reported by the CDC, perhaps due to overreporting on social media platforms. In contrast, the number of deaths reported in tweets (n=75) was comparable to the CDC estimates (n=85), suggesting an increased importance placed on COVID-19 deaths as the pandemic progressed.

**Table 4:** Comparison of infections and deaths among dental professionals with Centers for Disease Control and Prevention (CDC) data in the United States (2020).

Category	Reported through tweets	Based on CDC^a^ in 2020
Infections	117	98
Deaths	75	85

^a^CDC: Centers for Disease Control and Prevention.

## Discussion

### Principal Findings

Safeguarding health care workers is an essential component of any national strategy addressing a pandemic crisis, particularly as governments endeavor to enhance health care infrastructure to manage the escalating demand for urgent medical services. If unchecked, escalating infection and mortality rates among dental health care professionals will impede a nation’s ability to respond effectively to COVID-19, with potentially profound and enduring consequences for health care delivery. This impact is particularly severe in health care systems already facing challenges such as shortages of trained personnel, skilled labor migration, and geographic mal-distribution, issues that predate the pandemic [28]. The current study delves deep into the social behavior of populations using social media during a pandemic and provides comprehensive coverage of the data available internationally. It was found that the most widely spread information in relative tweets was about aerosol as a transmission route of COVID-19, similar to a previous study on COVID-19–related tweets on Weibo, the most popular internet-based microblog platform in China [7]. Public concern about the risks of spreading coronavirus by aerosols was diffused earlier than scientific publications, with the first tweet on Weibo in January 2020, while the first study reporting the possibility of COVID-19 transmission via aerosol-generating dental procedures was published in February 2020 [3], alongside another study confirming SARS-CoV-2 existed in saliva, which was also published in February 2020 [1]. This suggests that social media can serve as a useful tool to communicate health-related information.

### Demographics of COVID-19 Cases

As the aim of this study was to investigate the use of X to relay information related to the infection or death of dental professionals or patients due to COVID-19, the keywords used clearly reflected that, and strict inclusion and exclusion criteria were applied. Accordingly, a total of 5641 eligible tweets were retrieved, of which 1583 were deemed relevant. Of the relevant tweets, 3011 described infections at dental practices, where the majority (1168) of infection cases were reported among dentists, 134 among dental staff, and 41 among patients. The higher number of infections among dentists is not surprising, as it can be attributed to the proximity of the dentist to the patient during the dental procedures and the greater chance of contracting the infection from seeing many patients throughout the day.

**Figure 8:**
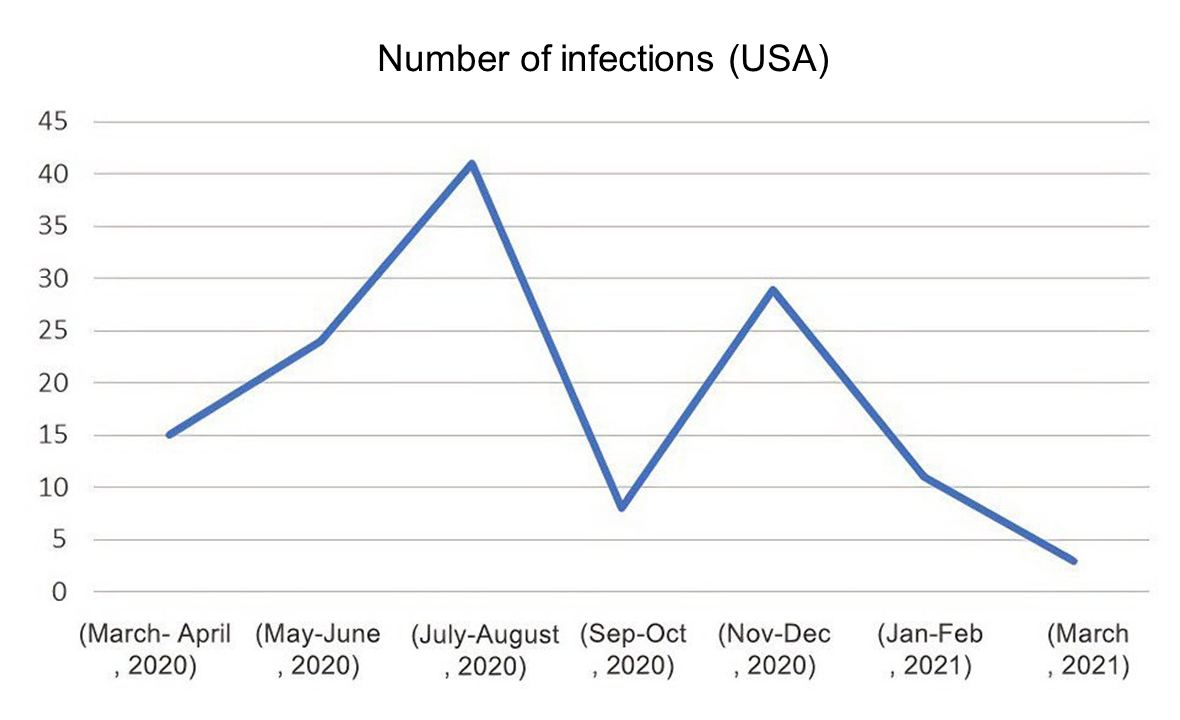
Diagram illustrating COVID-19 infections reported in the United States during the pandemic.

The most common countries reporting COVID-19 infections on X in the current study were the United States (Figure 8), India, and Canada. This could be related to the population density in these countries. In addition, since Twitter was originally launched in the United States, the largest number of users also happens to be from there, with around 73 million users, followed by Japan and India [12]. The age range of reported infection cases was 20-51 years. Statistics have shown that 59.2% of X users are between 25 and 49 years [12]. However, the average retirement age for dentists in the United States is 69, which is considered higher than other countries when compared with the United Kingdom, for example, where dentists retire at an average age of 59 years [29,30]. Therefore, the age range was not unexpected in the current findings.

According to the reported death cases in tweets, 600 deaths from COVID-19 were described by X users, of which 253 were among dentists, 22 among dental staff, and 7 among patients, again showing a higher number of affected dentists compared with dental personnel and patients. Of these, 98 were reported to be male and 32 females. The higher number of infected females, while the higher number of male deaths, correlates with other studies describing how more men die from COVID-19, whereas women’s general health is only being negatively affected [31-33]. Another possible reason for the higher number of reported male deaths in tweets could be that over 60% of Twitter users are male, and therefore, they are tweeting more about COVID-19 than females [12].

### Geographical Distribution of Tweets

On March 6 and 7, 2020, the Pacific Dental Conference took place in Vancouver. Nearly 15,000 people who attended the conference were told to self-isolate immediately after by British Columbia’s provincial health officer, who notified that multiple cases of COVID-19 had been traced to the event. Data in our study showed a surge of tweets disseminating information about the event (Multimedia Appendix 1), with some users communicating the news, for example:

FIFTEEN THOUSAND dentists were at a conference in Vancouver March 6-7, and within a week, 4 of attendees had tested positive for #Covid19.

Others showed concern about how the situation was dealt with:

A B.C. dentist who attended a Vancouver conference at the center of a coronavirus outbreak has died of suspected COVID-19 complications. The death is raising questions over why patients at the dentist's practice were not alerted over his illness.

Such phenomena suggest that social media engages public attention during outbreaks of pandemics [7]. Some tweets identified specific areas where dental practices have been affected by COVID-19. This can be a useful tool to monitor health disasters during unprecedented times [34,35]. Example tweets were:

Uh oh, a dentist in Arizona tested positive for Covid19. And I heard of another dental office in New Jersey had a provider test positive too” and “Dentist & Hospital Staff test COVID-19 positive in Odisha.

Results from a Canadian cohort research that sought to determine the prevalence of COVID-19 among dentists working in the community were included in the literature. The study findings show that 5 dentists are reporting COVID-19 infections for every 100,000 people per day in Canada. This incidence rate was concluded after a 188-day follow-up with participants. To put it another way, they found that 1.08% of dentists in Canada tested positive for COVID-19 during the study period from July 2020 to February 2021. This indicates that the dental community and the general public can feel reassured by the low infection rate observed [36].

In 2021, India set global records for daily COVID-19 infections, repeatedly surpassing the 300,000 tally previously set by the United States, due to the ease in restrictions. The peaks in tweets found in the current study were also influenced by any crisis taking place. One can argue that while using X as a source of information has some advantages, the disadvantage might be the lack of critical assessment and evaluation of health-related information [37].

### X and Policy Makers

Most policy makers and health care providers rely on the CDC and the WHO to be notified of disease outbreaks or to be informed of new infectious diseases, including evolving epidemiological data [7,38]. However, at the time of the outbreak, the novel COVID-19 infection was not under control, and many researchers were attempting to gain a better understanding of the evolution of the virus [39,40]. COVID-19 became a critical concern for public health, and it is the duty of policy makers to prevent the spread of the virus and ensure public safety. A public health emergency was declared in many countries worldwide, with governments facing the challenging responsibility of balancing public health protections and civil liberties [37]. Social media could be an effective early predictor of the spread of a particular disease, enabling government health sectors to identify potential and high-risk outbreak areas. Accordingly, they could prepare in advance for epidemic prevention and formulate new public health policies at an early stage [15].

As a link was established between practicing dentistry and the risk of COVID-19 transmission between dentists and patients, tweets from dental professionals at the end of January 2020 urged to stop dental services. That was followed by public health policy decisions in some countries to suspend dental practices at the beginning of February [7]. This demonstrates the influence social media has on policy makers and the evidence they can obtain to make health policy decisions [41]. An example of a tweet by a patient who was objecting to dentists practicing during the pandemic stated,

Dentists should be ashamed of themselves for staying open and inviting the spread of this disease 😷 #dentists.

While on the other hand, dental professionals tweeted,

#Dentists are highly exposed to diseases and infection compared to other professions. We should delay non-urgent dental care”, or “Trying to hang in. 1st

In the current study, it was observed that some users were tweeting warning messages, such as

#Coronavirus: Indonesia sees cases surge as death toll among doctors’ mounts. The pandemic has claimed the lives of at least 22 doctors, six dentists and 10 nurses, amid concerns about access to personal protective equipment”, or “Yesterday, Dean of Cairo dentist University got coronavirus. Please elderly doctors and doctors with known chest or immunity conditions, stay home, use video conference. DAILY HOSPITALS SANITATION”.

It is not unusual for the public to display engagement and protective behavior, similar to what has been reported during previous pandemics [17,42,43].

### Limitations

A few limitations of this study should be acknowledged. First, while in-depth statistical analysis of the characteristics of the tweets could have been performed, the focus of the study was on descriptive analyses of reported COVID-19 infections and deaths in dental practices, including patients, dentists, and dental personnel, to identify the impact of COVID-19 on dentistry. Second, in the geographic analysis of tweets, many tweets did not provide location information and had to be excluded. Therefore, affecting the overall number of confirmed cases and the objectivity of results to a certain extent. In addition, some countries do not use X as their method for communicating news, therefore, limiting data collection from those regions. Finally, since the name “Coronavirus” was not officially public until February 2020.

### Conclusion

Data suggests that platform X has the potential as a real-time surveillance tool for tracking COVID-19 cases and fatalities within the dental profession. By analyzing social media data, it provides insights into the geographic and demographic impact of the pandemic on dental practitioners and patients, highlighting the platform’s usefulness as an early indicator of disease spread. The study’s findings suggest that, beyond COVID-19, similar methods could help health care providers detect infection trends early and implement preventive measures. This research advocates for incorporating social media into public health monitoring, which could enable more rapid responses to emerging health crises. Moving forward, establishing clear guidelines for social media–based surveillance, including standards for data accuracy, privacy, and effective communication, could strengthen the reliability of such tools, making the health care sector better equipped to manage future pandemics and health threats.

## Appendix

Multimedia Appendix 1 Diagram showing high COVID-19 infections in Canada between March and -April 2020. X and Policy makers.

## Abbreviations

API: application programming interface
CDC: Centers for Disease Control and Prevention
WHO: World Health Organization
