# Correction: Infoveillance of COVID-19 Infections in Dentistry Using Platform X: Descriptive Study

**DOI:** 10.2196/86469

**Published:** 2026-03-31

**Authors:** Alghalia Al-Mansoori, Ola Al Hayk, Sharifa Qassmi, Sarah M Aziz, Fatima Haouari, Tawanda Chivese, Faleh Tamimi, Alaa Daud

**Affiliations:** 1 College of Dental Medicine QU Health Qatar University Doha Qatar

In “Infoveillance of COVID-19 Infections in Dentistry Using Platform X: Descriptive Study” [1], the authors noted that one name is missing from the Acknowledgments section.

In the Acknowledgments section, the sentence originally appeared as:

The authors would like to thank Dr Rula Chami for her contribution to this research. The study received no funding.

This sentence now reads:

The authors would like to thank Dr Rula Chami and Layan Mohamed for their contribution to this research. The study received no funding.

The correction will appear in the online version of the paper on the JMIR Publications website, together with the publication of this correction notice. Because this was made after submission to PubMed, PubMed Central, and other full-text repositories, the corrected article has also been resubmitted to those repositories.
