# Supplementary figures and images for: Infoveillance of COVID-19 Infections in Dentistry Using Platform X: Descriptive Study
Source: J Med Internet Res. 2025 Apr 3;27:e54650. doi: 10.2196/54650 (PMC12006773; doi:10.2196/54650)

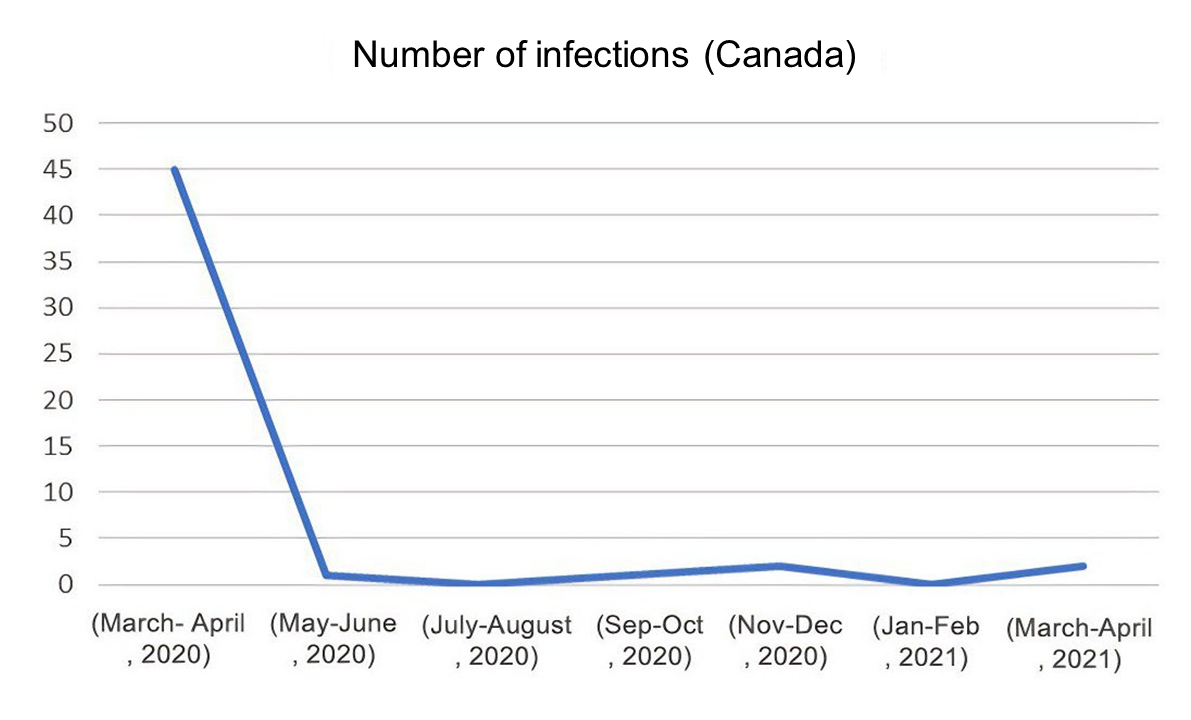

Supplement: Multimedia Appendix 1 [file jmir_v27i1e54650_app1.png]
